# Attitudes and Barriers Toward Antiretroviral Therapeutic Drug Monitoring Among Infectious Disease Providers

**DOI:** 10.3390/medicina61030544

**Published:** 2025-03-20

**Authors:** Hongmei Wang, Cecilia M. Torres, Thomas P. Giordano, Bich N. Dang, Dong Liang

**Affiliations:** 1 Joan M. Lafleur College of Pharmacy and Health Sciences, Texas Southern University, Houston, TX 77004, USA; cecilia.torres@tsu.edu; 2 Section of Infectious Diseases, Department of Medicine, Baylor College of Medicine, Houston, TX 77030, USA; tpg@bcm.edu (T.P.G.); bndang@bcm.edu (B.N.D.)

**Keywords:** attitude, barriers, perception, HIV, antiretroviral, ART, providers, therapeutic drug monitoring (TDM)

## Abstract

*Background/Objectives*: Effective HIV treatment and prevention rely heavily on patient adherence to the prescribed regimen. Therapeutic drug monitoring (TDM), which involves measuring medication concentrations in blood circulation, offers an objective method to evaluate toxic or ineffective drug levels. TDM is not routinely used in HIV treatment in clinical practice. Therefore, the purpose of this study is to survey infectious disease providers’ attitudes and barriers toward therapeutic drug monitoring for antiretroviral therapy in people living with HIV. *Materials and Methods*: A 15-item online survey was distributed to infectious disease providers in the Greater Houston area, including physicians, pharmacists, and mid-level practitioners involved in HIV patient care. The survey was disseminated via the Houston Citywide Infectious Disease Provider Network and the Houston AIDS Education and Training Center. The survey employed close-ended questions to evaluate providers’ attitudes, perceptions, practices, and barriers toward antiretroviral drug level monitoring. Responses were recorded using a five-point Likert scale. Demographic characteristics and information regarding research involvement were collected to contextualize the findings. The survey results were analyzed using descriptive statistics, with categorical variables expressed as frequencies and percentages using SAS software. *Results*: A total of 139 responses were received, with 89 participants meeting the inclusion criteria; the majority were female (62.9%), nearly half were aged 34 or younger (53.4%), 50% were physicians and 36.3% pharmacists, and most worked in hospitals (52.3%) or clinics (35.2%). The findings demonstrate participants’ predominantly positive attitudes toward TDM. Nearly 70% agree (agree or strongly agree) that TDM will be helpful and will positively impact improving drug efficacy and medication adherence. The results revealed barriers to implementing TDM, including a lack of evidence supporting TDM’s impact on HIV outcomes, and the absence of clinical guidelines. The results indicated that >90% were ambivalent or agreed that there was not enough evidence to support the use of TDM, and nearly all recognized that the guidelines do not endorse it or did not know if they do not endorse it. *Conclusions*: This study highlighted positive attitudes and significant barriers to implementing therapeutic drug monitoring, including a lack of evidence supporting TDM’s impact on HIV outcomes and the absence of clinical guidelines supporting TDM’s widespread use. The findings emphasize the need for clinical trials and longitudinal studies to establish definitive evidence on the effectiveness of TDM in improving HIV treatment outcomes.

## 1. Introduction

HIV remains a major global public health challenge, with approximately 39.9 million people living with HIV (PLWH), 1.3 million new infections, and 630,000 deaths reported in 2023 [1]. The Joint United Nations Programme on HIV/AIDS (UNAIDS) aims to end AIDS by 2030 through the 95-95-95 targets: 95% of PLWH identified, 95% on ART, and 95% achieving viral suppression [2]. Despite advancements in treatment and prevention through highly active antiretroviral therapy (ART), its effectiveness relies heavily on patient adherence [3,4,5]. High adherence to ART is essential for achieving undetectable viral loads, improving immune function, and reducing HIV transmission [6,7,8]. Research indicates that Black or African Americans face more significant barriers to adherence and HIV care compared to their White counterparts [9,10], further compounding health disparities. In addition, a critical factor in the success of ART is ensuring appropriate drug exposure, which can be influenced by various factors, including drug–drug interactions; physiological changes during pregnancy; altered drug absorption in certain populations such as children, adolescents, and individuals with malabsorption disorders; as well as organ dysfunction [11,12,13,14,15]. Maintaining appropriate drug levels is essential for ART effectiveness. Supratherapeutic drug levels can lead to toxic reactions, while subtherapeutic levels may compromise treatment efficacy and contribute to viral resistance. Inadequate ART exposure, whether due to poor adherence or altered drug metabolism, not only harms individual health but also facilitates the development and transmission of drug-resistant HIV strains, posing risks to public health [16,17].

Therapeutic drug monitoring (TDM), which involves the measurement of medication concentrations in biological fluids, offers a promising approach to optimize ART by guiding dose adjustments in these populations and ensuring treatment outcomes and toxic reaction management in PLWH [18,19,20]. TDM has been shown to help maintain plasma drug levels within the therapeutic range, reducing the risk of treatment failure due to subtherapeutic levels [21,22,23], which can lead to viral suppression failure and resistance mutations. Conversely, excessive plasma concentrations of certain antiretroviral drugs, particularly protease inhibitors and non-nucleoside reverse transcriptase inhibitors, have been linked to adverse effects, such as hepatotoxicity, neuropsychiatric symptoms, and metabolic disorders [24,25,26]. Additionally, TDM has been shown to help manage drug–drug interactions among different ART drugs or ART and other drug classes [27]. Special populations, including individuals with co-morbidities, pregnant individuals, and children, may require tailored TDM strategies to account for varying pharmacokinetics and potential drug interactions. For example, physiological changes during pregnancy, such as reduced intestinal motility, increased hepatic enzyme activity, and an enhanced glomerular filtration rate, can alter drug metabolism and necessitate dosage adjustments to maintain efficacy while minimizing maternal and fetal health risks. To address these concerns, twice-daily dosing of darunavir/ritonavir or raltegravir is recommended during pregnancy, as once-daily administration may result in trough drug concentrations (levels just before the next dose) falling below the desired therapeutic range [28]. Similarly, children, particularly neonates and preterm infants, metabolize drugs differently than adults, requiring careful consideration of dosing regimens and monitoring to prevent toxicity or subtherapeutic exposure. Case reports have highlighted the need for individualized TDM strategies in neonatal intensive care settings. In two cases, preterm infants receiving intermittent raltegravir dosing exhibited significantly delayed drug elimination, requiring less frequent administration to avoid excessive drug accumulation [29,30]. By implementing TDM strategies tailored to these special populations, healthcare providers can optimize treatment outcomes while fostering a more supportive environment encouraging patients to engage with their care plans.

Despite TDM’s potential benefits in improving treatment outcome and toxic reaction management, its implementation in clinical practice encounters several significant challenges. Healthcare providers often face time constraints and limited resources and facility capabilities, which impede their ability to effectively incorporate TDM into patient care [31]. Additionally, there is a recognized need for increased education and training among providers regarding managing medication adherence, including the utilization of TDM. Recent studies have highlighted these barriers, for instance, technological and logistical constraints in adopting TDM [32,33], critical gaps in provider training [33,34], and guideline availability [35]. However, concerns about the accurate interpretation of the results underscore the need for standard TDM protocols and training [36,37]. Despite these advancements, there remains a limited understanding of HIV providers’ attitudes and perceived barriers toward ART TDM. Assessing provider perspectives is essential for informing the development of effective research initiatives and implementation strategies.

Therefore, this study aims to investigate the attitudes and perceived barriers of HIV care providers toward the implementation of TDM for ART treatment. We hope to provide actionable insights for developing targeted interventions, such as protocols, provider training, and technological advancements. Ultimately, the findings aim to guide the integration of TDM into routine clinical practice, fostering improved adherence outcomes and advancing the personalized care of individuals living with HIV.

## 2. Materials and Methods

This study was a cross-sectional study consisting of a one-time electronic survey conducted in February 2024 among infectious disease providers caring for people living with HIV in Houston, Texas.

### 2.1. Study Design

A 15-question survey was adapted from similar studies based on a literature review [29,38] and conducted by an online survey (Qualtrics survey tool) [30]. The questionnaire was first piloted among two pharmacy students at Texas Southern University. After 1 round of modification, the questions were finalized. An email invitation to complete the survey was distributed to infectious disease providers in the Greater Houston area, including physicians, pharmacists, and mid-level practitioners (nurse practitioners and physician assistants) involved in HIV patient care. The survey was disseminated via the Houston Citywide Infectious Disease Provider Network and the Houston AIDS Education and Training Center. Selection criteria for survey participants included the following: a. professional roles (physicians, pharmacists, nurse practitioners, and physician assistants actively involved in HIV patient care); b. practicing within the Greater Houston area; c. regular interaction with patients receiving HIV treatment and/or management; d. willingness to provide informed consent and complete the survey in its entirety.

The survey employed close-ended questions to evaluate providers’ attitudes, perceptions, practices, and barriers related to medical research. A Likert 5-point scale ranging from strongly disagree to strongly agree was used to specify the level of agreement or disagreement with attitudes and barriers to using TDM. Additionally, demographic characteristics and information regarding research involvement were collected to contextualize the findings. All responses were anonymous. This study was registered with and approved by the Texas Southern University Institutional Review Board (#1821A). Consent for participation was obtained through the online survey tool’s starting page, where individuals received background information on the survey.

### 2.2. Statistical Analysis

The overall survey results are presented using descriptive statistics. All variables were categorical and expressed as frequencies and percentages. The data collected using a 5-point Likert scale were analyzed quantitatively by assigning numerical values to each point on the scale (e.g., 5 for “strongly agree” and 1 for “strongly disagree”). When the field “other” provided the option to enter free text, answers were coded and sorted where possible. Only completed responses were analyzed, and the number of answers to each question was counted. The data were analyzed using SAS software (version 9.4).

## 3. Results

### 3.1. Demographic Characteristics

**Figure 1:**
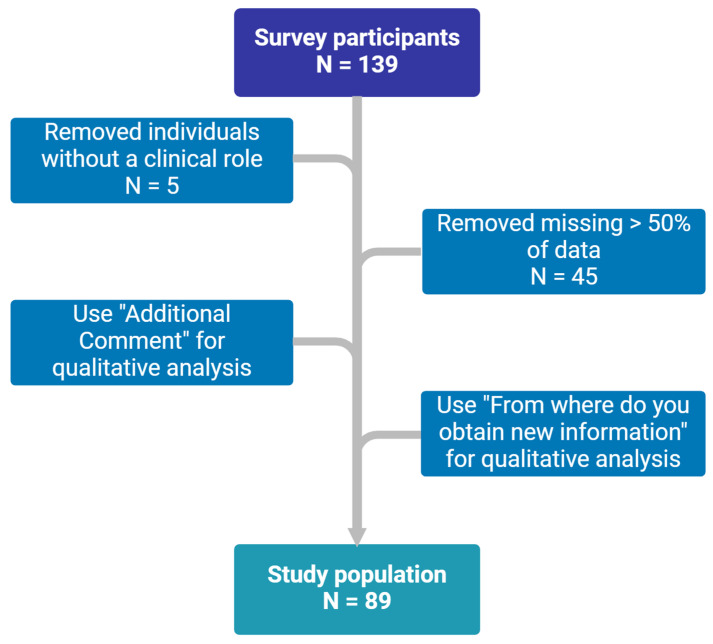
Study flowchart (created in BioRender. https://BioRender.com/z62c236 (accessed on 29 November 2024).

Responses were received from 139 participants, of whom 89 met the inclusion criteria, as summarized in Figure 1. The baseline characteristics of the participants are presented in Table 1. Most were female (62.9%), and nearly half (53.4%) were 34 or younger. More than half of the participants (52.3%) worked in hospitals, 35.2% in clinics, 10.2% in academia, and 2.3% in public health departments. The sample reflected diverse professional roles, with 50% being physicians, 36.4% pharmacists, and 10.2% mid-level practitioners such as nurse practitioners and physician assistants. There were three clinical roles categorized as “Other” that were laboratory technicians. Although they did not meet the original selection criteria, they should be considered stakeholders, and their opinions are highly valuable for TDM clinical practice. Therefore, we included their survey results for data analysis for this study.

In terms of experience in infectious disease practice, approximately one-third of participants (34.8%) were still in training, 36.0% had 0–5 years of experience, 11.2% had 6–10 years of experience, 10.1% had 11–19 years of experience, and 7.9% had over 20 years of experience. Regarding their patient populations, 56.2% reported that more than 10% of their patients were living with HIV, with 27.0% indicating that over 50% of their patient population consisted of individuals with HIV. Monthly encounters with HIV patients varied, with 60.7% seeing more than 5 HIV patients and 21.4% managing more than 30. The viral suppression rates among patients on ART were noteworthy, with 21.4% achieving suppression rates exceeding 95%. However, 67.4% of participants lacked access to TDM for antiretrovirals with 32.6% reporting that they were able to order TDM for antiretrovirals.

### 3.2. Attitude About TDM

**Figure 2:**
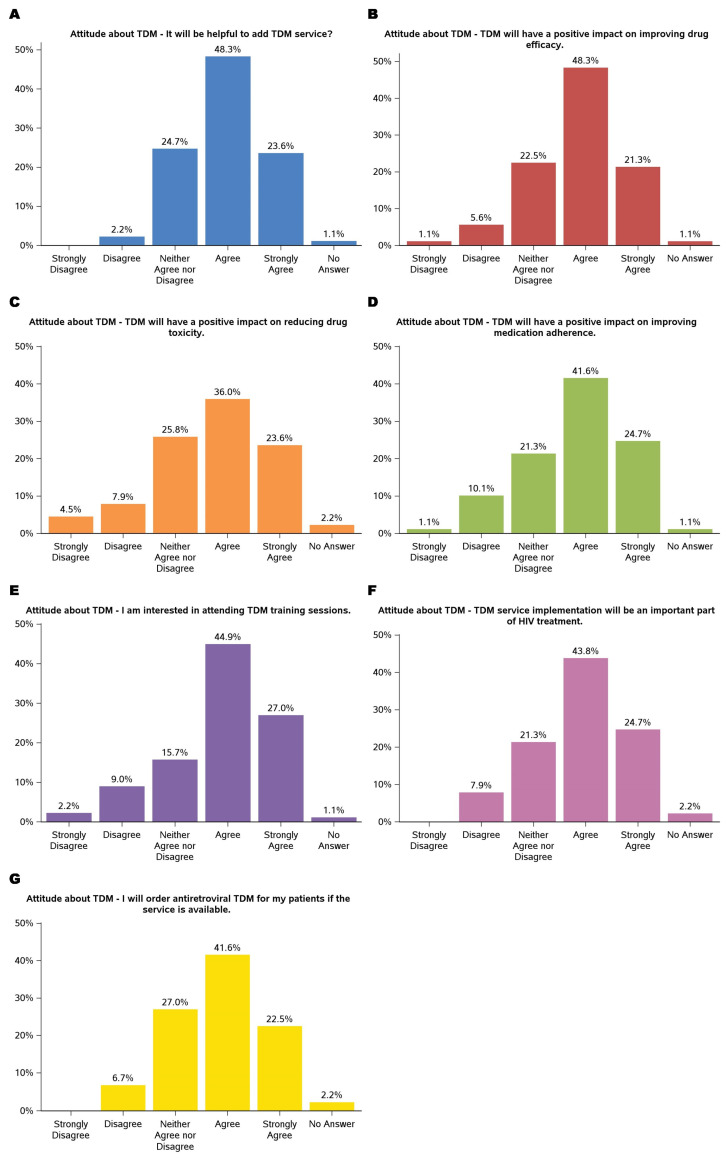
Attitudes about TDM: helpful to add TDM service (**A**); positive impact on improving drug efficacy (**B**); positive impact on reducing drug toxicity (**C**); positive impact on improving medication (**D**); interested in attending TDM training sessions (**E**); service implementation will be an important part of HIV treatment (**F**); will order antiretroviral TDM for patients if the service is available (**G**).

The findings demonstrated participants’ predominantly positive attitudes toward TDM. The results are summarized in Figure 2. Nearly 72% agreed (agreed or strongly agreed) that adding TDM services would be helpful. Similarly, the majority acknowledged TDM’s potential to enhance clinical outcomes, with 48.3% agreeing and 21.3% strongly agreeing that it could improve drug efficacy, 59.6% (36.0% agreed and 23.6% strongly agreed) recognizing its role in reducing drug toxicity, and 66.3% (41.6% agreed and 24.7% strongly agreed) believing it could positively impact medication adherence. These results suggested that TDM is a valuable tool for optimizing drug therapy and addressing challenges in patient management, particularly for complex treatment regimens such as ART.

Participants also expressed enthusiasm for integrating TDM into their practice and professional development. A large portion (44.9% agreed and 27.0% strongly agreed) showed interest in attending TDM training sessions, highlighting the need for education on implementing this service. Additionally, most of the participants (43.8% agreed and 24.7% strongly agreed) agreed that TDM would play an essential role in HIV treatment, and 64.1% (41.6% agreed and 22.5% strongly agreed) indicated they would order TDM for their patients if the service were available. While a minority expressed neutral or dissenting views, these findings underscore a strong inclination toward adopting TDM as a clinical practice component, emphasizing its potential to improve patient outcomes.

### 3.3. Barriers to TDM

**Figure 3:**
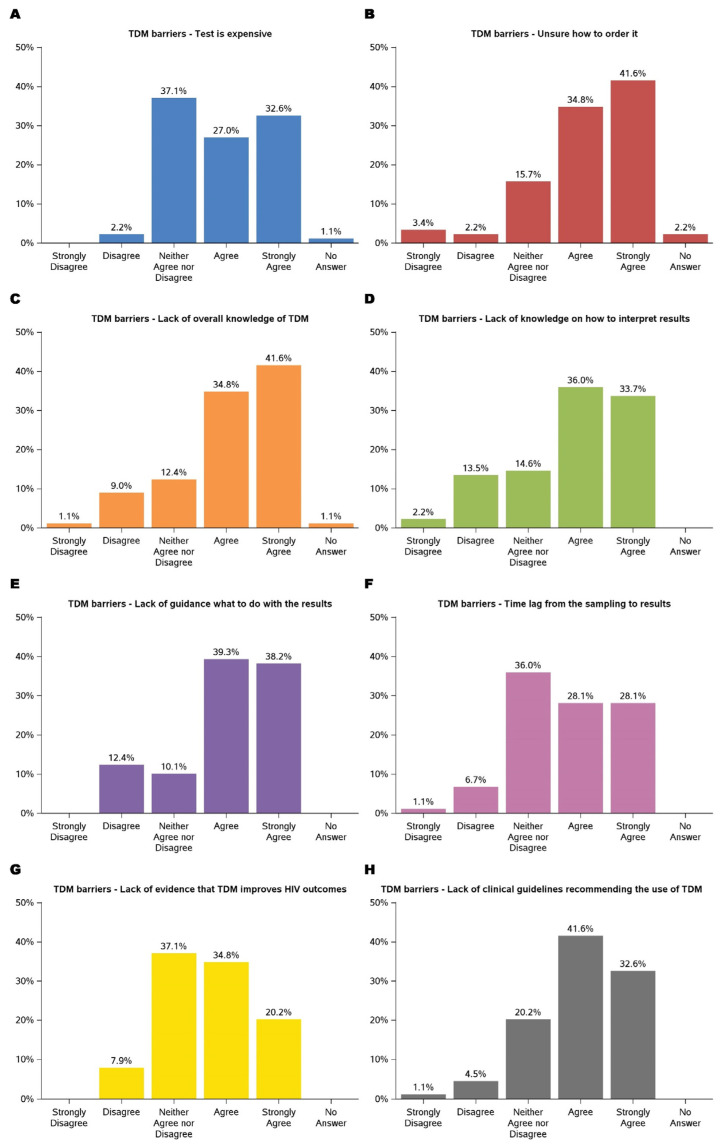
TDM barriers: test is expensive (**A**); unsure how to order it (**B**); lack of overall knowledge of TDM (**C**); lack of knowledge on how to interpret results (**D**); lack of guidance on what to do with the results (**E**); time lag from the sampling to results (**F**); lack of evidence that TDM improves HIV outcomes (**G**); lack of clinical guidelines recommending the use of TDM (**H**).

Barriers to implementing TDM were multifaceted, as shown in Figure 3. The key barriers were the lack of evidence and guidelines supporting TDM in improving HIV treatment outcomes. Participants expressed concerns about the limited availability of evidence demonstrating TDM’s impact on HIV outcomes, with over 90% either ambivalent or agreeing that there was not enough evidence to support its use. Similarly, nearly 95% of participants recognized that existing guidelines either did not endorse TDM or were uncertain about their stance. These challenges underscore the necessity for robust clinical data and guideline development to support the integration of TDM into routine HIV care.

Financial constraints were among the most frequently cited challenges, with 27.0% agreeing and 32.6% strongly agreeing that cost posed a significant barrier. Participants expressed concerns about the affordability of TDM services, particularly in resource-limited settings, which could limit accessibility and sustainability. Additionally, procedural uncertainties emerged as a prominent barrier, with 76.4% agreeing (34.8% agreed and 41.6% strongly agreed) that a lack of knowledge about ordering TDM services hindered its implementation. These findings highlighted the need for financial strategies and accessible procedural resources to support TDM adoption.

Knowledge gaps also presented substantial challenges. Approximately 33.7% strongly agreed and 36.0% agreed that interpreting TDM results was difficult. Nearly 80% of participants agreed that there is a lack of guidance on using TDM results, such as addressing medication adherence, pharmacogenetic differences that affect metabolism, pharmacokinetics changes in special populations, and significant drug–drug interactions. These findings align with previous barriers related to the lack of evidence and guidelines supporting TDM. To address these concerns, further research is necessary to generate high-quality evidence, develop evidence-based guidelines, and enhance provider training to facilitate the effective implementation of TDM in HIV care.

## 4. Discussion

TDM may be a valuable tool in the management of antiretroviral treatment. The findings of this study shed light on the significant barriers and overall positive attitudes toward TDM in the management of ART for individuals living with HIV. Consistent with prior research, procedural uncertainties emerged as a prominent barrier [32,33,34]. The financial concerns are compounded by procedural barriers, including knowledge gaps in ordering, interpreting, and acting on TDM results. This underscores the need for targeted educational initiatives and resources to support healthcare providers in effectively integrating TDM into clinical practice. Furthermore, the time delays associated with obtaining TDM results were cited as a significant logistical challenge, potentially impacting timely clinical decision-making and patient care. This may change quickly because many clinical labs are now equipped with Liquid chromatography-tandem mass spectrometry (LC-MS/MS) and data will be generated much faster.

A notable finding was the lack of evidence supporting TDM use in HIV management, which many providers identified as a critical impediment. These findings highlight the varied practice experiences and underscore the importance of targeted support for managing HIV patients effectively. This aligns with earlier studies emphasizing the necessity of standardized protocols to ensure the appropriate and consistent application of TDM [31,32]. The findings of this study highlight the critical gaps in evidence and guidelines supporting the use of therapeutic drug monitoring (TDM) in HIV treatment. While TDM has demonstrated utility in managing drug exposure and toxicity in specific populations, such as pregnant individuals, children, and patients with complex drug interactions [11,12,13,14,15], its role in optimizing ART remains uncertain. The survey results underscore the prevailing uncertainty among providers, with a majority recognizing the potential benefits of TDM but also acknowledging the lack of definitive clinical outcomes supporting its widespread use.

The lack of robust clinical trials evaluating the impact of TDM on HIV outcomes further complicates its adoption in clinical guidelines. While small-scale studies suggest potential benefits [33,34], large-scale longitudinal research is needed to establish its efficacy in improving viral suppression, reducing drug resistance, and enhancing overall treatment success. At present, the World Health Organization (WHO) has not issued formal recommendations on the role of TDM in HIV treatment [35]. Both the European AIDS Clinical Society (EACS) and U.S. Department of Health and Human Services (DHHS) guidelines acknowledge that TDM may be considered in patients with virologic failure [36,37]. The EACS guidelines further extend the role of TDM to special populations, adherence assessments, second-line ART, and very young children [37]. While routine TDM is not currently recommended, it may be beneficial in specific cases such as suspected malabsorption; drug–drug interactions; toxicity management; and in special populations including children, pregnant individuals, the elderly, and patients with good adherence but suboptimal treatment responses [38]. The results of our study emphasize the urgent need for further research to generate high-quality evidence that can inform guideline development and support the integration of TDM into routine HIV care. Until such evidence is available, the adoption of TDM will likely remain limited, constrained by provider uncertainty and the absence of clear clinical recommendations.

Despite these barriers, the overall attitudes toward TDM were predominantly positive. Most participants recognized its potential to enhance drug efficacy, reduce toxicity, and improve medication adherence. However, an aspect arose from our data: while participants showed enthusiasm for TDM’s potential benefits, a substantial proportion also agreed that there is a lack of robust evidence demonstrating TDM’s positive impact on clinical outcomes. This dichotomy highlights the need for further research to substantiate TDM’s efficacy and ensure its credibility among healthcare providers.

This study has several strengths, including its sample population of clinicians and pharmacists from a large metropolitan area with a high number of HIV patients and well-established healthcare facilities, such as those within the Texas Medical Center. The diverse professional backgrounds of respondents enhance the generalizability of the findings. However, there are also limitations. This study was a preliminary survey rather than a controlled study comparing the effectiveness of TDM, limiting the ability to draw causal inferences. Additionally, the sample size was relatively small and included a limited percentage of infectious disease specialists. The sample size also did not allow for stratified analyses based on clinical roles (e.g., physicians, advanced practice providers, pharmacists) or practice settings (e.g., inpatient vs. outpatient), which may have provided further insights into the variation in attitudes toward TDM.

In conclusion, providers agree that TDM could play a role in clinical care and that they would order it if the service were available, affordable and timely. However, providers cite a lack of evidence that TDM improves HIV outcomes in routine care, and a lack of guidelines recommending TDM in routine HIV care (outside of specific populations). Future efforts should focus on determining the impact of TDM on clinical outcomes so that evidence-based guidelines can address TDM.

## Figures and Tables

**Table 1:** Characteristics of participants (n = 89).

	Characteristics	Number (Percent)
Gender	Male	32 (36.0%)
	Female	56 (62.9%)
Age	≤34 years	47 (53.4%)
	35–49 years	34 (38.6%)
	50–64 years	6 (6.8%)
	≥65 years	1 (1.1%)
Employment	Hospital	46 (52.3%)
	Clinic	31 (35.2%)
	Public health department	2 (2.3%)
	Academia	9 (10.2%)
Clinical role	Physician	44 (50%)
	Physical assistant	3 (3.4%)
	Nurse practitioner	6 (6.8%)
	Pharmacist	32 (36.4%)
	Other	3 (3.41%)
Years in infectious disease practice	Still training	31 (34.8%)
	0–5 years	32 (36.0%)
	6–10 years	10 (11.2%)
	11–19 years	9 (10.1%)
	≥20 years	7 (7.9%)
% of patients with HIV	<10%	32 (36.0%)
	11–25%	18 (20.2%)
	26–50%	8 (9.0%)
	>50%	24 (27.0%)
	Unsure	7 (7.9%)
number of HIV patients/month	<5	28 (31.5%)
	5–10	10 (11.2%)
	11–20	14 (15.7%)
	20–30	11 (12.4%)
	>30	19 (21.4%)
% of patients with HIV achieving viral suppression on antiretrovirals	<50%	11 (12.4%)
	50–75%	12 (13.5%)
	76–85%	24 (27.0%)
	86–95%	23 (25.8%)
	>95%	19 (21.4%)
Able to order TDM of antiretroviral drugs	No	60 (67.4%)
	Yes	29 (32.6%)
TDM orders within 1-year period for antiretroviral drugs (N = 29)	<1 time per year	16 (55.2%)
	1–3 times per year	7 (24.1%)
	3–5 times per year	1 (3.5%)
	5–10 times per year	-
	>10 times per year	5 (17.2%)

Footnote: others under clinical roles were laboratory technicians. The denominator in the “Number of TDM orders within 1-year period for antiretroviral drugs” is 29 because it is contingent on the ability to order TDM drugs. The “Ability” variable has 60 no and 29 yes responses.

## Data Availability

The data presented in this study are available at the request of the corresponding author due to the potential risks of identifying individuals in the sensitive demographic categories included in the survey.

## References

[1] HIV.gov Global HIV/AIDS Overview. 17 December 2024. https://www.hiv.gov/.

[2] UNAIDS The Path That Ends AIDS: UNAIDS Global AIDS Update. July 2023. https://www.unaids.org/.

[3] Giordano T.P., Suarez-Almazor M.E., Grimes R.M. (2005). The population effectiveness of highly active antiretroviral therapy: Are good drugs good enough?. Curr. HIV/AIDS Rep..

[4] Bangsberg D.R., Perry S., Charlebois E.D., Clark R.A., Roberston M., Zolopa A.R., Moss A. (2001). Non-adherence to highly active antiretroviral therapy predicts progression to AIDS. AIDS.

[5] Parienti J., Fournier A.L., Cotte L., Schneider M., Etienne M., Unal G., Perré P., Dutheil J., Morilland-Lecoq E., Chaillot F. (2021). Forgiveness of Dolutegravir-Based Triple Therapy Compared with Older Antiretroviral Regimens: A Prospective Multicenter Cohort of Adherence Patterns and HIV-RNA Replication. Open Forum Infect. Dis..

[6] Eisinger R.W., Dieffenbach C.W., Fauci A.S. (2019). HIV Viral Load and Transmissibility of HIV Infection: Undetectable Equals Untransmittable. JAMA.

[7] Manalel J.A., Kaufman J.E., Wu Y., Fusaris E., Correa A., Ernst J., Brennan-Ing M. (2024). Association of ART regimen and adherence to viral suppression: An observational study of a clinical population of people with HIV. AIDS Res. Ther..

[8] Komandt M., Canfield S., Lengel M., Gilmore V., Kilcrease C. (2023). Correlation between medication adherence using proportion of days covered and achieving viral suppression in patients living with HIV. JMCP.

[9] Burton K.L., Ritchwood T.D., Metzger I.W. (2023). Structural Racism and Racial Trauma Among African Americans at Elevated Risk for HIV Infection. Am. J. Public Health.

[10] Laurencin C.T., Murdock C.J., Laurencin L., Christensen D.M. (2018). HIV/AIDS and the African-American Community 2018: A Decade Call to Action. J. Racial Ethn. Health Disparities.

[11] Goodlet K.J., Zmarlicka M.T., Peckham A.M. (2019). Drug–drug interactions and clinical considerations with co-administration of antiretrovirals and psychotropic drugs. CNS Spectr..

[12] Nhean S., Tseng A., Back D. (2021). The intersection of drug interactions and adverse reactions in contemporary antiretroviral therapy. Curr. Opin. HIV AIDS.

[13] Lim S.Y., Pettit R.S. (2019). Pharmacokinetic considerations in pediatric pharmacotherapy. Am. J. Health-Syst. Pharm..

[14] Collins I.J., Turkova A. (2023). A step closer to optimal ART for all children. Lancet HIV.

[15] Jhajra S., Singh S., Holm K.A., Faqi A.S., Sahi J. (2012). Influence of changes in physiology, transporters, and enzyme expression on disposition and metabolism of drugs during pregnancy and clinical implications. Encyclopedia of Drug Metabolism and Interactions.

[16] Kim J., Lee E., Park B., Bang J.H., Lee J.Y. (2018). Adherence to antiretroviral therapy and factors affecting low medication adherence among incident HIV-infected individuals during 2009–2016: A nationwide study. Sci. Rep..

[17] Ekstrand M.L., Shet A., Chandy S., Singh G., Shamsundar R., Madhavan V., Saravanan S., Heylen E., Kumarasamy N. (2011). Suboptimal adherence associated with virological failure and resistance mutations to first-line highly active antiretroviral therapy (HAART) in Bangalore, India. Int. Health.

[18] Scibona P., Cruz C.V., Lopardo G., Gimenez M.I., Frecha C., Rodriguez L.F., Rolon M.J., Cecchini D., Cordova E., Abela C. (2018). Individualized Antiretroviral Therapy. Impact of pharmacogenetic and therapeutic drug monitoring in the safety and efficacy of first line antirretroviral therapy in patients with HIV infection. Preliminary report. Int. J. Infect. Dis..

[19] Aarnoutse R.E., Schapiro J.M., Boucher C.A.B., Hekster Y.A., Burger D.M. (2003). Therapeutic Drug Monitoring. Drugs.

[20] Burger D.M. (2002). Criteria for Therapeutic Drug Monitoring in HIV. https://hospitalpharmacyeurope.com/news/editors-pick/criteria-for-therapeutic-drug-monitoring-in-hiv/.

[21] Perry C. (2023). Monitoring Drug Therapy: Importance, Methods, and Challenges. J. Dev. Drugs.

[22] Milone M.C., Shaw L.M. (2014). Breaking the Therapeutic Drug Monitoring Logistics Barrier. Clin. Chem..

[23] Chatjaroenpat S., Chuenmueang C., Jaisue S. (2024). A cross-sectional study of the current situation with therapeutic drug monitoring in Thailand: Requirements, challenges and the role of educational institutions. Pharm. Educ..

[24] Bjørlykke K.H., Jahnsen J., Brynskov J., Molander P., Eberhardson M., Davidsdottir L.G., Sipponen T., Hjortswang H., Goll G.L., Syversen S.W. (2023). Therapeutic drug monitoring in inflammatory bowel disease: Implementation, utilization, and barriers in clinical practice in Scandinavia. Scand. J. Gastroenterol..

[25] DeArmond P.D., Bunch D.R., Dasgupta A. (2024). Chapter 10—Therapeutic drug monitoring of antiretroviral drugs for the management of human immunodeficiency infection. Therapeutic Drug Monitoring.

[26] Preskorn S.H. (2021). Charting and Handling Therapeutic Drug Monitoring Results: How they Differ from Most Laboratory Results. J. Psychiatr. Pract..

[27] Hazarika I. (2015). Therapeutic Drug Monitoring (TDM): An Aspect of Clinical Pharmacology and Pharmacy Practice. Res. Rev. J. Pharmacol..

[28] Selinger C., Carbonell J., Kane J., Omer M., Ford A.C. (2021). Acceptability of a ‘treat to target’ approach in inflammatory bowel disease to patients in clinical remission. Frontline Gastroenterol..

[29] Grossberg L.B., Papamichael K., Feuerstein J.D., Siegel C.A., Ullman T.A., Cheifetz A.S. (2017). A Survey Study of Gastroenterologists’ Attitudes and Barriers Toward Therapeutic Drug Monitoring of Anti-TNF Therapy in Inflammatory Bowel Disease. Inflamm. Bowel Dis..

[30] (2018). Qualtrics in Academic Research. https://www.qualtrics.com/blog/citing-qualtrics/.

[31] Archibald T.L., Murrell D.E., Brown S.D. (2018). Chromatographic methods in HIV medicine: Application to therapeutic drug monitoring. Biomed. Chromatogr..

[32] van Gelder T. (2023). The Appropriately Designed TDM Clinical Trial: Endpoints, Pitfalls, and Perspectives. Ther. Drug Monit..

[33] Buzibye A., Musaazi J., von Braun A., Nanzigu S., Sekaggya-Wiltshire C., Kambugu A., Fehr J., Lamorde M., Gutteck U., Muller D. (2019). Antiretroviral concentration measurements as an additional tool to manage virologic failure in resource limited settings: A case control study. AIDS Res. Ther..

[34] Li N., Zheng H., He W., He X., Li R., Cui W., Yang W., Dong X., Shen Z., Zheng Y. (2024). Treatment outcomes amongst older people with HIV infection receiving antiretroviral therapy. AIDS.

[35] World Health Organization (WHO) (2021). Consolidated Guidelines on HIV Prevention, Testing, Treatment, Service Delivery and Monitoring: Recommendations for a Public Health Approach. https://www.who.int/publications/i/item/9789240031593.

[36] HIV.gov (2024). Guidelines for the Use of Antiretroviral Agents in Adults and Adolescents with HIV. https://clinicalinfo.hiv.gov/en/guidelines/hiv-clinical-guidelines-adult-and-adolescent-arv/whats-new.

[37] European AIDS Clinical Society (EACS) (2023). EACS Guidelines for the Management of People Living with HIV in Europe. https://www.eacsociety.org/guidelines/eacs-guidelines/.

[38] Rindi L.V., Zaçe D., Compagno M., Colagrossi L., Santoro M.M., Andreoni M., Perno C.F., Sarmati L. (2024). Management of low-level HIV viremia during antiretroviral therapy: Delphi consensus statement and appraisal of the evidence. Sex. Transm. Infect..

